# Spectrum of Beta-Thalassemia Mutations in Potential Carriers with Microcytic Hypochromic Anemia from Mazandaran and Golestan, Northern Provinces of Iran

**DOI:** 10.1155/2024/8664803

**Published:** 2024-01-30

**Authors:** Seyed Saeed Mousavi, Hossein Karami, Ahmad Tamadoni, Hassan Mahmoudi, Ramin Shekarriz, Rita Siami, Mohammad Bagher Hashemi-Soteh

**Affiliations:** ^1^Novin Genetic Diagnostic Laboratory, Farah Abad Boulevard, Sari, Iran; ^2^Department of Pediatrics Hematology & Oncology, Thalassemia Research Center, Faculty of Medicine, Mazandaran University of Medical Sciences, Sari, Iran; ^3^Non-Communicable Pediatric Diseases Research Center, Health Research Institute, Babol University of Medical Sciences, Babol, Iran; ^4^The Clinical Research Development Unit of Amirkola Children's Hospital, Babol University of Medical Sciences, Babol, Iran; ^5^Department of Hematology and Oncology, Gastrointestinal Cancer Research Center, Mazandaran University of Medical Sciences, Sari, Iran; ^6^Ghaemshahr Health Center, Mazandaran University of Medical Sciences, Sari, Mazandaran, Iran; ^7^Immunogenetic Research Center, Molecular and Cell Biology Research Center, Biochemistry and Genetic Department, Medical Faculty, Mazandaran University of Medical Sciences, Sari, Iran

## Abstract

**Introduction:**

*β*-Thalassaemia is the most common genetic disorder and is considered as a major public health concern in Iran. Different countrywide studies have shown a heterogeneous mutational basis of *β*-thalassaemia with different frequencies in each area. This study is aimed at investigating the common and rare mutations in Mazandaran and Golestan, northern provinces of Iran.

**Methods:**

5425 microcytic and hypochromic individuals were investigated from Mazandaran and Golestan provinces. From these, 1323 beta carrier or affected individuals were selected where 938 persons were from Mazandaran and 385 people were from Golestan province, respectively.

**Result:**

53 different mutations were identified, IVSII-1 (G>A) was the most common (59.14%) followed by Cd 22/23/24 (-7 bp) (5.34%), Cd 8 (-AA) (4.93%), Cd30 (G>A) (4.00%), and IVSI-5 (G>C) (3.70%) with a total of 77.11% in Mazandaran Province, respectively. In Golestan Province, IVSI-5 (G>C) was the most frequent (44.62%) followed by IVSII-1 (G>A) (27.18%), Cd 15 (TGG>TAG) (4.36%), Fr 8/9(+G) (3.85%), and Cd 8(-AA) (2.05%) with a total of 82.06%, respectively. From the 53 different mutations, 22 numbers have been observed in both provinces. Two deletions of the beta gene named Sicilian and Asian-Indian have been detected in Mazandaran with a frequency of 0.72% each.

**Conclusion:**

The 53 different mutations identified in this study were the most ever reported mutations in the country. Due to diversity of different ethnic groups, there are many varieties of mutation in beta globin gene in Iran. It could be assumed that both founder effect and natural selection caused by migration from neighboring areas have complemented each other to produce the high frequency of unique alleles within each region.

## 1. Introduction

*β*-Thalassaemia is the most common genetic disorder and is considered as a major public health concern in Iran. It is estimated that up to 7% of the world population are carriers of hemoglobin diseases, such as *β-* or *α*-zero thalassemia (*α*0-thal), or hemoglobin S, C, D Punjab, or E [1]. The prevalence of *β*-thalassemia is high in the Middle East, Southeast Asia, and Mediterranean Region and increasing in nonendemic regions including North America and Western Europe due to migration pattern [2]. The gene frequency of *β*-thalassaemia is high in Iran and varies considerably from area to area. It is believed that the highest rate is around the Caspian Sea with up to 8% [3–5]. Nearly 2800 patients are register in health care services in Mazandaran and about 402 patients in Golestan Province according to Thalassemia Registry in Mazandaran Province (http://thr.mazums.ac.ir). According to previous studies, the frequency of carriers is between 2 and 9.5% in different provinces, of which Mazandaran and Golestan are among the highest number of carriers with about 8% [6, 7]. One study in Golestan Province showed a frequency of 9.8% for beta-thalassemia carriers [8]. The high prevalence of *β*-thalassaemia in this region is believed to be related to endemic malaria [9]. National thalassemia screening program that is a premarriage screening started since 1995, which initially uses a cell blood count (CBC) and hemoglobin electrophoresis tests from the couple before marriage, to determine beta-thalassemia carriers as well as anemic people due to hemoglobin defects such as sickle cell anemia, hemoglobin E, and alpha thalassemia [10, 11].

Prenatal diagnosis (PND) requires a genetic test to identify defects or mutations in parents followed by mutation detection in fetus after pregnancy [12]. Performing PND for the fetus and medical abortion considerably reduced the incidence of thalassemia in Iran, including Mazandaran and Golestan provinces [6, 7, 11, 13].

A long list including 950 different mutations is reported in hemoglobin database HbVar or “A Database of Human Hemoglobin Variants and Thalassemias” from different countries to date (http://globin.bx.psu.edu/hbvar/menu.html). Also, several countrywide studies have shown that the mutational basis of *β*-thalassaemia in Iran is heterogeneous, and more than 50 different mutations are detected across different geographic regions of Iran [14–19]. The spectrum of these mutations is variable in each area and depends on geographic regions, ethnic group, and immigration. Few reports are available regarding the spectrum and frequency of mutations causing *β*-thalassaemia in Mazandaran and Golestan provinces to date [20–22]. This study is aimed at investigating the common mutations in the region. This data will help us to update our knowledge about common as well as rare mutations in *HBB* gene in northern provinces of the country. It also will be helpful for *β*-thalassaemia carriers screening, genetic counseling, and prenatal diagnosis of the disease in these area as well as in the country.

## 2. Materials and Methods

### 2.1. Study Population

As part of the national thalassemia screening program, high-risk couples or couples who were already detected as beta-thalassemia carrier referred to a designated local laboratory for genetic tests. It is a retrospective and cross-sectional study, with nonprobability and consecutive sampling between 2008 and 2021 among the individuals who were referred to a diagnostic genetic laboratory. People with beta globin gene mutation are included, and those with alpha globin gene mutation or without mutation were excluded from this study.

People were first screened by CBC (cell blood count) and hemoglobin electrophoresis. Those who had reduced MCV (MCV ≤ 80 fl) or MCH (MCH ≤ 27 pg/cell) [23] were subjected to further study and referred to a local genetic laboratory as a routine national services [10]. Also, for the HbA2 level, concentration more than 3.5% was considered as beta-thalassemia carrier [10, 24], and HbF hemoglobin normal level in adults is considered from 0.5% to 1.5% [24, 25]. From 5425 microcytic and hypochromic people referred to “Novin Genetic Diagnostic Laboratory” during 2008 to 2021, 1323 persons were beta carrier or affected individuals, who were referred from Mazandaran or Golestan provinces (Table 1). All individuals were informed about this research, and written consent was obtained from each patient; meanwhile, name and personal data of the participants will remain confidential.

### 2.2. Genomic DNA Extraction

A 5 to 10 ml venous blood was obtained from each subject and stored in Na-EDTA tube at -25°C until processing. Lymphocytic genomic DNA was extracted by Nucleon BACCII method [26] followed by DNA concentration measurement using *NanoDrop*™ *2000*/*2000c* Spectrophotometers (Thermo Fisher Scientific, USA).

### 2.3. ARMS-PCR Amplification for Common Mutations

**Figure 1:**
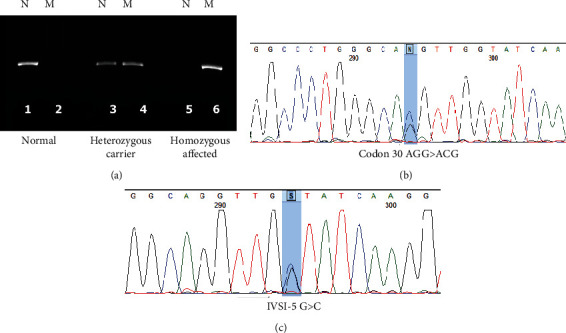
(a) An example of ARMS-PCR for mutation detection. Lanes 1 and 2 show a normal sample for IVSII-1 (G>A) in which only normal (N) tube amplified, lanes 3 and 4 show a heterozygous mutant sample in which both normal (N) and mutant (M) tubes amplified, and lanes 5 and 6 show a homozygous mutant sample in which only mutant (M) tube amplified. (b) DNA sequence chromatogram shows nucleotide change G>C in a position c.92 or codon 30 (AGG>ACG) in HBB gene in a heterozygous carrier person. (c) A nucleotide transition G>C in intron 1, position c.92+5 or IVSI-5 in HBB gene in a heterozygous carrier person.

The specific primers were used to amplify each specific mutation separately. The ARMS-PCR was applied for the detection of 10 common mutations previously reported including IVSII-1 (G>A), IVSI-5 (G>C), Cd 22/23/24 (del GAAGTTGGT), Cd 30 (G>C), Cd 8 (-AA), Fr 8/9 (+G), Cd 44 (-C), IVSI-I (G>A), Cd 36/37 (-T), and IVSI-25bp del [19, 27]. Briefly, three specific primers were used to genotype the mentioned mutations, two specific forward primers for normal and mutant alleles, respectively, and one common reverse primer. For each sample, two tubes were used as M and N. A 25 *μ*l master mix PCR containing 11 *μ*l “Taq DNA Polymerase 2x Master Mix RED” (Amplicon, Denmark), 0.6 *μ*l of forward and 0.6 *μ*l reverse primer, and 2 *μ*l DNA template up to 25 *μ*l dH2O were applied in each tube. The PCR reactions were carried out with the following conditions: 93°C, 40 seconds; annealing temperature for 40 seconds; and 72°C 40 seconds for 30 cycles. PCR products were visualized on 1% agarose gel. Results were evaluated as normal when only N tube amplified, homozygous mutant (MM) when only M tube amplified, and heterozygous normal/mutant (NM), when both tubes amplified (Figure 1(a)).

### 2.4. DNA Sequencing

Samples that showed no mutations using conventional ARMS-PCR methods were subjected to further investigations using Sanger DNA sequencing method (Applied Biosystems, Waltham, USA). In brief, DNA sequencing method was used to screen entire coding and noncoding regions of the HBB gene using specific primers (Table 2) to find any point mutations or short deletion/insertions. A DNA sequence analysis software, Gene Runner (http://www.generunner.com), was applied along with using reference sequences from GenBank database. Finch TV, a DNA sequence chromatogram viewer software (Geospiza, Inc., USA), was also applied to view nucleotide changes.

### 2.5. Gap-PCR and Multiplex Ligation-Dependent Probe Amplification (MLPA)

Samples that showed no mutations using different methods were subjected for further study using Gap-PCR and the MLPA method (MRC-Holland, Netherland) to identify any potential large deletion or insertion [28]. Some frequent deletions including Hb Lepore, Hb Sicilian, and Hb Asian-Indian were detected using previously described Gap-PCR [29]. Subsequently, in those that mutation was not identified, MLPA assay using SALSA MLPA Probemix P102 HBB (MRC-Holland, Amsterdam, Netherlands) was applied searching for deletions or gene fusion within the HBB cluster [12, 28, 30].

## 3. Result

5425 microcytic and hypochromic people who were referred to the Novin Genetic Diagnostic Laboratory during 2008 to 2021 were investigated. In total, 4500 persons were from Mazandaran and 925 individuals were from Golestan Province, respectively. From these, 1323 beta carrier or affected individuals were selected where 938 persons were from Mazandaran (902 carrier and 36 affected) and 385 persons were from Golestan Province (380 carrier and 5 affected), respectively (Table 1). 53 different mutations were identified in this study (Table 3). From the 974 mutant chromosomes investigated in Mazandaran Province, IVSII-1 (G>A) was the most common mutation with a frequency of 59.14% followed by four mutations including Cd 22/23/24 (-7 bp) (5.34%), Cd 8 (-AA) (4.93%), C30 (G>A) (4.00%), and IVSI-5 (G>C) (3.70%), respectively. These 5 mutations represented 77.11% of the total mutations in Mazandaran Province (Table 3). From the 4500 individuals investigated from Mazandaran Province, 28 (0.62%) individuals were HbS patients including 27 heterozygous carrier and 1 homozygous and 2 sickle-thal affected, respectively. We also found 49 individuals (1.08%) with HbD Punjab (Cd 121 GAA>CAA) variant including two HbD homozygous in Mazandaran. Also, 5 individuals (0.11%) were identified with HbE (Cd 26 GAG>TAG) variant in Mazandaran population among the 4500 individuals.

In Golestan Province, IVSI-5 G>C was the most frequent mutation with the frequency of 44.62% followed by IVSII-1 (G>A) (27.18%), Cd 15 (TGG>TAG) (4.36%), Fr 8/9 (+G) (3.85%), and Cd 8 (-AA) (2.05%), respectively. These 5 mutations represented 82.06% of the total mutations in Golestan Province (Table 3). Also, among the 925 individuals, there were 2 (0.22%) carrier individuals for HbS hemoglobin and 28 individuals (3.03%) including 27 heterozygous and 1 homozygous for HbD Punjab (Cd 121 GAA>CAA) hemoglobin. Also, 4 beta-thalassemia carrier had HbD hemoglobin as well. Also, 3 individuals (0.32%) with HbE (Cd26 GAG>TAG) variant were identified in Golestan Province, respectively.

Among the beta-thalassemia carriers that were studied in this research, some showed normal HbA2 level between 1 and 3.5%.  Table 4 shows 92 persons from Mazandaran and 20 from Golestan Province with normal level of HbA2 hemoglobin. Although some previously known mutations like -101, UTR+22, are IVSI-6 are listed among these mutations, some beta-zero mutations like IVSII-1 and IVSI-5 can also be seen among this list (Table 4).

## 4. Discussion

Beta-thalassemia is a heterogeneous and diverse disease from molecular point of view. In HbVar hemoglobin database (http://globin.bx.psu.edu/hbvar/menu.html), 950 variant alleles have been reported in beta globin gene to date [31]. A small number of thalassemia mutations are predominant in most parts of the world, and the most common ones tend to be those that are geographically the most widespread [32]. For instance, six common mutations including IVSII-745 (C>G), IVSI-110 (G>A), IVSI-6 (T>C), IVSI-1 (G>A), IVSII-1 (G>A), and C39 (C>T) represent about 90% of all mutations in the Mediterranean area [33], and four alleles account for about 91% of the mutations in China and Southeast Asia [34]. Iran with a population of approximately 85 million represents a highly heterogeneous gene pool and mutation spectrum due to geographical, cultural, and ethnical diversity [14, 19, 35].

Beta-thalassemia gene mutation distribution and frequency are well studied and reported in different provinces in Iran. For example, a study in Hormozgan Province (south of Iran) reported IVSI-5 (G>C) (69%) as a most frequent, followed by IVSII-1 (G>A) (9.6%) [36]. Other study in Azerbaijan Province (northwest of the country) showed that IVSII-1 (G>A) (21%) and IVSI-110 (G>A) (18%) were more frequent [37]. Kurdistan Province in the west also showed IVSII-1 (G>A) with 31% as the most frequent mutation [38]. In the southwest province of Khuzestan, IVSII-1 (G>A) with 34% was the most frequent, followed by Fr 8/9 with 17.3% [39]. A recent study on the central province of Isfahan showed that IVSII-1 (G>A) (27.9%) and Fr 36/37(-T) (19.7%) were the most frequent mutations, respectively [40]. IVSII-1 (G>A) is a common mutation in the Mediterranean area and was reported as the most predominant in three northern (Gilan, Mazandaran, and Golestan) and northwestern (Azerbaijan and Ardebil) provinces of Iran [20, 37].

In this study, 53 different mutations were identified. From these, 22 mutations have been observed in both provinces, including IVSII-1 (G>A), IVSI-5 (G>C), Fr8/9 (+G), IVSI-25 del, C8 (-AA), IVSI-130 (G>C), IVS-110 (G>A), and IVSI-1 (G>A) which were more frequent, respectively (Table 3). Except of these common, about 31 remaining are detected in either both or one province with a frequency of less than 1% (Table 3). While IVSII-1 (G>A) was the most common mutation in Mazandaran (59.14%), IVSI-5 (G>C) was the most prevalent in Golestan Province (44.62%) followed by IVSII-1 (G>A) (27.18%) instead. One new or unreported mutation also is identified in a carrier person from Golestan Province in this study, a base pair deletion in codon 80, c.243 C>-, and C80 (AAC>AA-). From the 5 most common mutations detected in the two provinces, 3 of them are the same (IVSII-1, IVSI-5, and Cd 8), but with different frequencies (Table 3).

**Figure 2:**
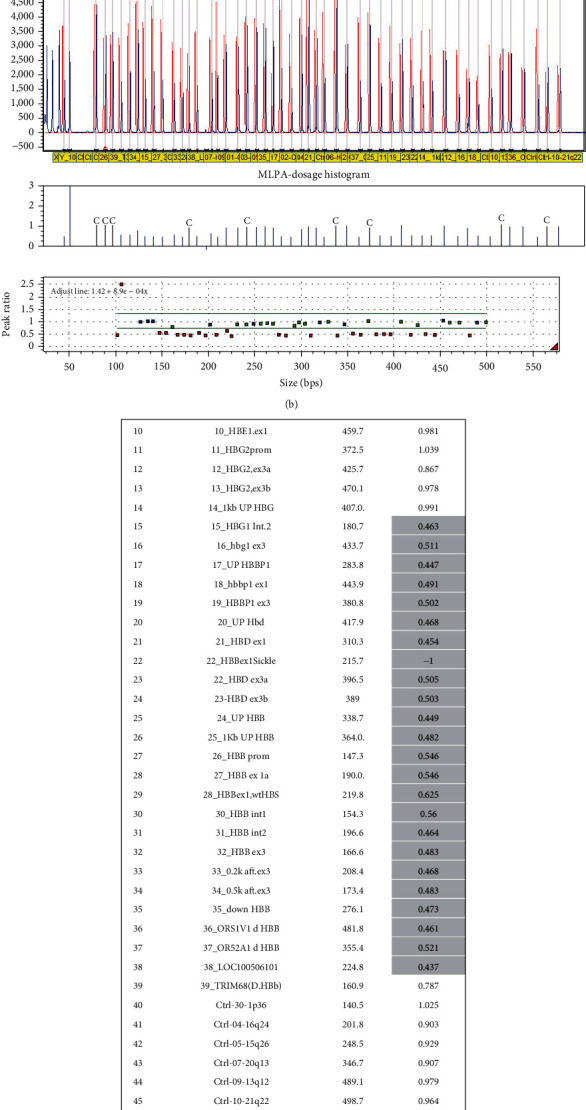
(a) Three different deletions found in this study. Sicilian and Asian-Indian deletions are common and reported before in Mazandaran Province. Also, a large and undefined deletion is identified with about 132.7 kb in beta-cluster genes toward TRIM68 gene in 3′. (b) MLPA result related to the 132.7 kb deletion. (c) Gray color in the column shows different probe deletions from intron2 of HGB1 gene to downstream of HBB gene (TRIM68 gene) with about 132.7 kb deletion in one carrier person.

In HBB gene, point mutations are prevalent, but still, there are some gene deletions which are detected and may be different from area to area. For instance, two gross deletions named Sicilian and Asian-Indian have been detected in Mazandaran Province with frequency of 0.72% and 0.72% in this study (Table 3) [29]. In addition, two other unknown deletions identified by MLPA method, deletion of exon 3 of beta globin gene, and a large deletion with about 132 kb length (from 5′, A-gamma gene, beta gene, and delta gene toward TRIM68 gene in 3′) were recognized in this study and need to be fully characterized later (Figure 2). The carrier person with large gene deletion had the following hematologic index: RBC: 6.35, Hbg: 17.1, MCV: 80.3, MCH: 26.9, HbA1: 79.4, HbA2: 4.4, and HbF: 16.2, respectively.

Different investigations revealed IVSI-5 (C>G) as the most prevalent mutation in the Middle East, India, and South and Southeast Asia before [32, 34, 41]. The IVSI-5 is the most common mutation among the people with Sistani and Baluch ethnic origin, from Sistan-Baluchestan Province, a southeastern part of Iran which has border with Pakistan [42]. Earlier study in this province reported IVSI-5 (G>A) (44.8%) as the most common mutation, similar to the frequency in Pakistan (53%) [43]. Because there was internal migration or relocation from Sistan and Baluchestan provinces to Golestan in recent decades and they are made up to 30% of the total population in Golestan Province now [44], so as a consequence, IVSI-5 is one of the frequent mutations in this province. Surprisingly, it is only found 3.7% in Mazandaran Province in the present study. Additionally, our results revealed the frequency of 3 hemoglobin variants, HbS, HbD, and HbE. In Mazandaran Province, frequency of HbS was 0.62%, HbD 1.08%, and HbE about 0.32%, respectively, in a total of 4500 people studied. In Golestan Province, frequency of HbS was found 0.22%, HbD 3.03%, and HbE about 0.32% in 925 individuals, respectively.

CBC index detection is the first line of laboratory examination to find carrier individual in Iranian national thalassemia program [23]. In the present study, carrier individuals with different mutations generally showed the mean MCV below 70 fl and MCH below 20 pg per cell, respectively (Table 5), but for the mutations such as -88, -101, 5′-UTR+22, CD27, CD126, IVSI-6, and IVSI-128, these values achieved were usually above 70 and 20, respectively. Moreover, for carriers of two deletion mutations, Sicilian and Asian-Indian, the MCV and MCH values were higher than 70 and 20 (Table 5). Furthermore, the HbA2 level in beta-thalassemia carrier is more than the normal level of 3.5% [23], but in this study, there were some carrier individuals with normal HbA2 level.  Table 4 lists a number of mutations including -101, 5′-UTR+22, CD27, CD126, IVSI-6, and IVSI-128. The Sicilian and Asian-Indian deletion mutations also have normal HbA2 level and increased HbF with averages of 6.85% and 6.4%, respectively (Table 4). Some beta-thalassemia carriers with common mutations such as IVSII-1 and IVSI-5 also show normal HbA2 level with unknown reason (Table 4). There were some limitations in this study. For instance, some families were not fully cooperative for additional investigations, so the blood samples from other family members were not available. Also because of the limitation in the budget, it was not possible to investigate and determine the exact length of the deletion in individuals who had new gene deletion.

## 5. Conclusion

The 53 different heterogeneous mutations identified among carriers and patients in this study were the most ever reported mutations both in the number and diversity compared with the previous reports in the country. Two previous studies in Mazandaran reported 17 and 25 different mutations in beta-thalassemia carrier and patients, respectively [20, 21]. Although the diversity and number of mutations reported here are higher, the frequency of common mutations was similar with the previous studies (Table 3). Five mutations were more common in both provinces in which three of them, IVSII-1, IVSI-5, and Cd 8, were similar between two provinces.

Due to presence and diversity of different ethnic groups in the country, there are many varieties in the molecular genetics of beta globin gene in Iran. The presence of different highly frequent mutants' alleles, along with some similarities in different geographical regions, is supporting a role of nonisolating genetically areas. It could be assumed that both founder effect and natural selection caused by migration from neighboring areas have complemented each other to produce the high frequency of unique alleles within each region.

## Figures and Tables

**Table 1:** Number of males and females as well as numbers of beta-thalassemia carriers and patients who were studied in this research (*n* = 1323).

Gender	Province				
	Mazandaran		Golestan		
	Carrier	Patient	Carrier	Patient	
Male	453	11	206	3	
Female	449	25	174	2	
Total	902	36	380	5	1323

**Table 2:** Specific primers for PCR sequencing of HBB gene.

HBB gene	Specific primer pairs	Annealing Tm	PCR product sizes
Exon 1 and 2	F: 5′ CTGAGGGTTTGAAGTCCAACTCC 3′	60°C	808 bp
	R: 5′ CTTCCACACTGATGCAATCATTC 3′		
Exon 3	F: 5′ CAATGTATCATGCCTCTTTGCAC 3′	60°C	580 bp
	R: 5′ GCACTGACCTCCCACATTCC 3′		

**Table 3:** Allele frequency and number of affected chromosomes by *β*-thalassaemia mutations in Mazandaran and Golestan provinces (*n* = 1364).

Mutation		Mazandaran	Percent	Golestan	Percent
-101 C>T	c.-151C>T	12	1.23	0	0
-88 C>A	c.-138C>A	0	0	6	1.54
-87 C>A	c.-137C>A	1	0.10	0	0.00
-30 T>C	c.-80T>C	2	0.21	0	0.00
-28 A>C	c.-78A>C	3	0.31	0	0.00
3-UTR-1570 T>C	c.^∗^96T>C	1	0.10	0	0.00
5-UTR-22 G>A	c.-29G>A	26	2.67	1	0.26
CD 5 (-CT)	c.17_18delCT	9	0.92	3	0.77
CD 8 (-AA)	c.25_26delAA	48	4.93	8	2.05
Fr 8/9 (+G)	c.27_28insG	13	1.33	15	3.85
CD 14/15 (+G)	c.45_46insG	0	0	1	0.26
CD 15 TGG>TAG	c.47G>A	2	0.21	17	4.36
CD 17 AAG>TAG	c.52A>T	0	0.00	1	0.26
CD 22/23/24 (-GAA GTT GGT)	c.68_74delAAGTTGG	52	5.34	1	0.26
CD 25/26 (+T)	c.78_79insT	1	0.10	0	0.00
CD 27/Hb Knossos GCC> TCC	c.82G>T	5	0.51	0	0.00
CD 30 AGG>ACG	c.92G>C	39	4.00	3	0.77
IVSI-1 G>A	c.92+1G>A	9	0.92	7	1.79
IVSI-2 T>C	c.92+2T>C	1	0.10	0	0.00
IVSI-5 G>C	c.92+5G>C	36	3.70	174	44.62
IVSI-6 T>C	c.92+6T>C	8	0.82	6	1.54
IVSI-25 del (-GTCTATTTTCCCACCCTTAGGCTGC)	c.93-22_95del	6	0.62	9	2.31
IVSI-110 G>A	c.93-21G>A	12	1.23	5	1.28
IVSI-128 T>G	c.93-3T>G	6	0.62	0	0.00
IVSI-129 A>G	c.93-2A>G	1	0.10	0	0.00
IVSI-130 G>C	c.93-1G>C	12	1.23	5	1.28
CD 36/37 (-T)	c.112delT	4	0.41	1	0.26
CD37/38/39 (-GACCCAG)	c.114_120delGACCCAG	0	0.00	1	0.26
CD39 CAG>TAG	c.118C>T	4	0.41	1	0.26
CD 41/42 (-TTCT)	c.126_129delCTTT	1	0.10	0	0.00
CD 44 (-C)	c.135delC	9	0.92	4	1.03
CD 56 (GGC>CGC)	c.169 G>C	1	0.10	0	0.00
CD 77 CAC>GAC	c.232C>G	1	0.10	0	0.00
CD 80 AAC>AA-	c.243 C>-	0	0.00	1	0.26
CD 81 CTC>-TC	C.245-246delTC	0	0.00	1	0.26
CD 80/81 (-C)	C.243-244delC	0	0.00	3	0.77
CD 82/83 (-G)	c.251delG	8	0.82	3	0.77
CD101 GAG>TAG	c.304G>T	1	0.10	0	0.00
IVSII-1 G>A	c.315+1G>A	576	59.14	106	27.18
IVSII-1 G>C	c.315+1G>C	2	0.21	0	0.00
IVSII-2 T>G	c.315+2T>G	3	0.31	0	0.00
IVSII-654 C>T	c.316-197C>T	2	0.21	0	0.00
IVSII-745 C>G	c.316-106C>G	21	2.16	4	1.03
IVSII-848 C>A	c.316-3C>A	1	0.10	1	0.26
IVSII-850 G>T	c.316-1G>T	8	0.82	0	0.00
Sicilian	g.64336_77738del13403	7	0.72	0	0.00
Asian-Indian	g.50509_83170del32662	7	0.72	0	0.00
CD 123 ACC>AAC	c.371C>A	1	0.10	0	0.00
CD 123/124/125 deletion	c.370_378delACCCCACCA	0	0.00	1	0.26
CD 126 GTG>GGG	c.380T>G	9	0.92	0	0.00
CD 146 CAC>CTC	c.440A>T	1	0.10	0	0.00
Delta-beta-gamma deletion	Delta-beta-gamma deletion	1	0.10	0	0.00
Exon 3 deletion	Exon 3 deletion	1	0.10	1	0.26
		974	100	390	100

**Table 4:** List of mutations that the HbA2 level among heterozygous carrier individuals were normal (1 to 3.5%). Also, mean and standard deviations of cell blood count (CBC) index from these individuals are shown.

Mutation	*n*	RBC	MCV	MCH	HB	HbA2	HbF
IVSII-1 G>A	22	5.6 + 1.6	61.0 + 5.9	19.2 + 5.5	10.4 + 3.7	1.4 + 1.5	1.5 + 1.1
.-101 C>T	3	5.3 + 0.02	79.5 + 1.4	26.0 + 1.8	2.9 + 0.3	2.9 + 0.3	0.6 + 0.4
.-87	1	4.41	87.1	26.8	11.8	2.1	0.9
3-UTR+1570	1	5.3	70.5	22.1	11.6	2.6	
5-UTR+22	13	5.3 + 0.6	69.3 + 14.8	23.3 + 2.0	11.8 + 3.9	2.8 + 0.4	0.7 + 0.5
C82-83	1	4.94	64	18.8	9.3	2.7	
CD101	1	6.11	79.5	24.5	15	3.2	
CD123	1	4.7	79.6	25.7	12.1	3.4	1.6
CD126	8	5 + 0.4	77.4 + 3.8	25.0 + 1.2	12.4 + 1.1	2.9 + 0.5	0.4 + 0.3
CD15	1	5.73	68.4	25.2	12.1	2.4	
CD22/23/24	2	5.2 + .02	64.6 + 8.8	20.4 + 2.3	11.2 + 0.5	1.6 + 2.3	1.3 + 0.3
CD27 Knossos	5	5.03 + 0.8	73.0 + 2.2	23.3 + 0.7	11.9 + 1.5	1.9 + 0.4	0.5 + 0.4
CD30	2	5.1 + 1.1	65.9 + 2	20.5 + 0.2	11.9 + 1.6	3.1 + 0.4	0.4 + 0.5
CD 5	1	5.6	57.9	19	10.6	2.6	0.5
CD 60	1	4.6	82.4	28.2	12.8	3.2	0.8
CD8	6	6.0 + 0.3	67.5 + 6.4	21.1 + 3.1	12.2 + 1.1	2.8 + 0.3	0.7 + 0.7
IVSI-1 G>A	2	5.1 + .1	75 + 6.2	22.5 + 3.6	2.7 + 0.14	2.7 + 0.1	0.7 + 0.1
IVSI-128	1	5.67	72.4	23	13.8	3.4	1
IVSI-129	1	5.37	76.5	23.9	12.7	3.5	0.1
IVSI-130	2	5.3 + 1.0	64.5 + 9.1	18.3 + 2.5	10.6 + 0.5	2.3	0.6
IVSI-5	4	5.0 +1.3	74.2 + 16.1	22.2 + 3.4	11 + 1.9	2.9 + 0.5	0.7 + 0.2
IVSI-6	1	6.3	71.4	20.1	12.7	2.5	1.2
IVSI-1	1	6.9	58	18	12.1	3.2	0.9
IVSI-5	13	5.7 + 0.6	66.0 + 5.5	19.9 + 1.9	10.3 + 3.2	3.0 + 0.3	1.0 + 2.6
IVSI-6	2	5.6 + 1.2	74.6 + 2.3	23.7 + 1.4	13.2 + 2.1	3.2 + 0.1	0.4 + 0.6
IVSII-1 G>C	2	5.5 + 1.7	69 + 7.1	21 + 4.2	11.3 + 1.6	3.2 + 0.3	2.2 + 0.1
Asian-Indian	6	5.6 + 0.8	71 + 1.6	23.3 + 1.0	13.3 + 1.7	2.6 + 0.8	6.4 + 9.4
Sicilian	6	5.9 + 0.6	70.1 + 6.1	21.5 + 2.3	12.8 + 1.6	2.5 + 0.2	6.9 + 5.2

**Table 5:** Mean of some cell blood count (CBC) index along with standard deviation (SD) for each of them; heterozygous carrier individuals with 29 more frequent beta gene mutations are listed.

Mutation	*n*	RBC	MCV	MCH	HB	HbA2	HbF
IVSII-1	624	5.99 ± 0.7	63.08 ± 4.22	19.54 ± 1.61	11.6 ± 1.3	5.18 ± 0.7	1.7 ± 1.8
CD22/23/24	47	5.9 ± 0.85	63.9 ± 5.7	19.5 ± 2.1	11.8 ± 1.2	4.9 ± 1.05	1.7 ± 3.3
CD30	40	5.8 ± 0.6	62.6 ± 3.6	19.2 ± 1	11.3 ± 1.3	4.8 ± 0.6	1.1 ± 0.8
CD8	56	5.9 ± 0.7	63.8 ± 4.1	19.5 ± 1.5	11.4 ± 1.2	4.8 ± 0.8	1 ± 1.2
Fr 8/9	26	5.8 ± 0.7	62.1 ± 2.7	18.2 ± 1.7	11.2 ± 1.5	5.3 ± 0.8	1.5 ± 1.2
IVSI-5	208	5.7 ± 0.7	64.8 ± 4.2	19.7 ± 1.3	11.5 ± 1.3	4.5 ± 0.7	1 ± 0.9
IVSI-25 del	15	5.9 ± 0.8	60.7 ± 3	18 ± 0.8	10.9 ± 1.3	4.7 ± 0.4	1 ± 0.5
CD39	5	5.9 ± 0.8	60.8 ± 2.9	18.5 ± 1	11 ± 1.4	4.77 ± 0.4	1.5 ± 0.4
CD44	11	5.98 ± 0.9	60.1 ± 5.5	18.5 ± 1.4	12 ± 1	5.4 ± 0.4	1.5 ± 1.1
IVSII-745	25	5.6 ± 1	64.8 ± 5.6	19.5 ± 1.2	10.9 ± 2	4.8 ± 1.2	0.7 ± 0.2
IVSI-1	16	5.9 ± 0.7	64.2 ± 5.3	19.5 ± 1.9	11.7 ± 1	4.4 ± 1	1 ± 0.7
IVSI-6	14	5.6 ± 0.6	71.5 ± 3.8	22.2 ± 1.8	12.6 ± 1.4	4 ± 0.8	0.9 ± 0.3
CD36/37	4	6.6 ± 1.5	61.1 ± 0.5	19 ± 0.2	12.4 ± 2.9	5.3 ± 05	1.1 ± 0.8
IVSI-110	17	5.8 ± 0.7	64.1 ± 4.1	19.9 ± 1.4	11.7 ± 1.5	4.9 ± 0.6	0.7 ± 0.3
CD5	12	5.9 ± 0.8	56.5 ± 4.5	19.2 ± 0.9	11.8 ± 1.5	4.9 ± 0.8	0.9 ± 0.9
CD15	18	5.9 ± 0.8	62.8 ± 3.18	19.2 ± 2.5	11.5 ± 1	4.5 ± 0.9	0.8 ± 0.4
.-88	6	5.8 ± 0.5	71 ± 1.6	23.5 ± 1.4	13.4 ± 1.4	5.5 ± 0.5	2.4 ± 0.4
.-101	9	5.2 ± 0.5	76 ± 8.2	25.2 ± 3	13.7 ± 1.2	4.11 ± 1.3	2.3 ± 3.8
5-UTR+22	26	5.4 ± 0.6	73.5 ± 3.9	23.4 ± 1.7	12.9 ± 1.3	3.3 ± 0.7	0.7 ± 0.3
IVSI-130	17	5.7 ± 0.7	63.1 ± 4.6	19.7 ± 1.6	11.4 ± 1.3	4.7 ± 0.8	0.8 ± 0.3
Asian-Indian	7	5.6 ± 0.8	70 ± 2	23 ± 1	13.3 ± 1.5	2.4 ± 1.1	5.6 ± 8.8
Sicilian	7	5.9 ± 0.5	70.3 ± 5.5	21 ± 2.1	12.7 ± 1.4	2.6 ± 0.5	7.1 ± 4.8
C82-83	10	4.9 ± 0.5	63.4 ± 3.3	18.8 ± 0.5	9.2 ± 1.2	4.2 ± 1.3	0.8
CD126	9	5 ± 0.3	78.6 ± 3.1	24.7 ± 1	12.2 ± 1	3.2 ± 0.6	0.6 ± 0.3
CD27 Knossos	5	5 ± 0.7	73 ± 2.1	23.3 ± 0.7	11.9 ± 1.4	1.9 ± 0.4	0.7 ± 0.2
CD80/81	3	6.1 ± 0.9	59 ± 1.3	18.3 ± 0.8	11.4 ± 1.8	4.7 ± 0.4	1 ± 0.1
IVSII+850	8	5.6 ± 0.8	61.8 ± 3.8	18.9 ± 1	10.7 ± 1.6	5.4 ± 0.8	0.8 ± 0.4
IVSI-128	6	6.1 ± 0.4	70.2 ± 3.2	22.6 ± 1.1	14 ± 0.9	4.4 ± 1	0.7 ± 0.3
IVSII-2	3	5.6 ± 0.6	63.9 ± 8	20.2 ± 3	11.4 ± 1.4	5.2 ± 0.2	0.6 ± 0.2

## Data Availability

The data used to support the findings of this study are included within the article. The data were supplied by a diagnostic laboratory and so cannot be made freely available. Requests for access to these data should be made to the corresponding author upon request.
